# Wave Energy Assessment at Valencia Gulf and Comparison of Energy Production of Most Suitable Wave Energy Converters

**DOI:** 10.3390/ijerph17228473

**Published:** 2020-11-16

**Authors:** Raúl Cascajo, Emilio García, Eduardo Quiles, Francisco Morant, Antonio Correcher

**Affiliations:** 1 Área de Ingeniería Naval y Oceánica, Universidad Politécnica de Madrid, 28040 Madrid, Spain; rcascajo@gmail.com; 2 Instituto de Automática e Informática Industrial, Universitat Politècnica de València, 46022 Valencia, Spain; egarciam@isa.upv.es (E.G.); fmorant@isa.upv.es (F.M.); ancorsal@ai2.upv.es (A.C.)

**Keywords:** wave energy, energy production, renewable energy, zero emissions port, wave energy converter

## Abstract

Seaports’ energy strategy should rely on the use of renewable energy. Presently, the share of renewable energy used by many of the ports worldwide is negligible. Some initiatives are in the process of implementation to produce some of the energy used by the Port of Valencia, one the largest ports in the Mediterranean Basin. Among these initiatives, a photovoltaic plant with an installed capacity of 5.5 MW is under a tendering process and the assessment studies for the deployment of three to five windmills are close to being finished. However, this is not enough to make it a “zero emissions port” as some of the energy demand would still be covered by fossil fuels. Therefore, we should consider clean alternative energy sources. This article analyses the wave energy resources in the surroundings of the Port of Valencia using a 7-year series of data obtained from numerical modelling (forecast). The spatial distribution of wave power is analysed using data from 3 SIMAR points at Valencia Bay and is compared to the data obtained by the Valencia Buoy I (removed in 2005). The obtained results are used to estimate the power matrices and the average energy output of two wave energy converters suitable to be integrated into the port’s infrastructure. Finally, the wave energy converters’ production is compared to the average amount of energy that is forecast to be obtained from other renewable sources such as solar and wind. Due to the nature of the Gulf’s wave climate (mostly low waves), the main conclusion is that the energy obtainable from the waves in the Valencia Gulf will be in correlation with such climate. However, when dealing with great energy consumers every source of production is worthwhile and further research is needed to optimize the production of energy from renewable sources and its use in an industrial environment such as ports.

## 1. Introduction

Some of the major seaports worldwide are developing energy policies aimed at the reduction of their greenhouse gas emissions. These policies consist of the reduction of the ports’ dependency from fossil fuels and the generation of energy from renewable sources, such as photovoltaic, wind, tidal or waves.

The level of maturity of wind and solar energy technologies makes these two energy sources the most feasible for installation in ports. However, if we consider solar energy, it must be taken into account that most of the port areas are very busy and subjected to the action of meteorological elements.

Wave energy seems to be one of the most promising alternatives to fossil fuels, the use of wave energy is estimated to increase significantly over the next few decades as wave energy converter (WEC) technology matures [1]. Moreover, according to a study by the European Technology and Innovation Platform for Ocean Energy, which was published in May 2020: “Europe’s ocean energy resource is considerable. By 2050, ocean energy can deliver 100GW of capacity- equivalent to 10% of Europe’s electricity consumption today. Flexible and predictable, ocean energy complements variable renewables such as wind or solar, that will dominate Europe’s electricity system in 2050. Ocean energy will play an important role in smoothing production peaks and balancing Europe’s electricity grid. By 2050, the ocean energy sector will employ 400,000 Europeans, ensuring a just transition to a decarbonized economy. Europe’s technological advantage in ocean energy will ensure European companies a large share of a strong global market, as they do on offshore wind. With zero carbon emissions, ocean energy will help tackle climate change and achieve a cleaner, more sustainable and more prosperous Europe.” 

While ocean wave energy conversion is still unproven in a commercial scale, significant advances in research, design and testing continue to be made [2] and a number of wave energy projects, almost all still in a pilot stage, are currently underway around the world [3,4].

According to some estimates [5], the potential of wave energy in the region of Valencia is 3.64 TWh/year gross or 0.58 TWh/year net.

Some WECs can be integrated into port infrastructure [6], thus obtaining significant savings from the power take off systems (PTO) as well as installation, operation and maintenance costs, while providing erosion protection to the marine structure. This means that wave energy could be part of the energy mix aiming to cover most of the energy needs of the port [7].

Research on wave energy resource assessment has been carried out in countries where great wave energy potential is expected [1]. Wave energy is particularly interesting for islands, which are usually highly dependent on external energy supply. Taking this into account, one could say that the ideal place for the exploitation of wave energy potential are islands located in large oceans.

Furthermore, ports need great amounts of energy from the shore and the fact that they have provided energy supplying infrastructure allows us to consider them as “energy islands”, making wave energy a good candidate to cover part of the ports’ energy needs.

Some ports worldwide have already integrated renewable energy generators in their facilities. Rotterdam, Antwerp, Amsterdam [8,9,10], amongst others, are examples of the integration of wind energy in ports, while Naples, Gibraltar, Mutriku and Civitavecchia [11,12], are examples of locations that have integrated wave energy converters into their infrastructure. At present, only at the location of the Port of Civitavecchia is the system not running. 

In relation to the Port of Valencia there are studies being developed by the Port Authority of Valencia (PAV) on the estimated energy production from renewable sources. The preliminary estimations provided a result of 8 GWh/year considering a total of 12,650 photovoltaic panels of 435 W each (size of each panel is around 2.1 square meters in size, resulting in overall space of 26,565 square meters required for the solar power station) on the roof of a new vehicle storage silo, covering almost 10% of the total consumption of the port of Valencia. In the case of the wind, the result obtained for this resource in the studied area is 5.19 m/s, with the predictions from the Spanish Energy Institute (IDAE) at the Valencia location being between 5.0 and 5.5 m/s at 100 m above the sea level [13]. Considering three wind turbines of 4.8 MW of nominal power, a total energy production of 26.63 GWh/y is estimated. This estimation would cover around 50% of the energy consumed by the Port of Valencia [7]. 

The aim of this paper is the evaluation of the wave energy resource at the Bay of Valencia, near the Port of Valencia. Once the wave energy resource has been estimated, two suitable WECs for ports are selected in order to estimate the energy production simulating their installation within the port’s existing infrastructure and general comparison to the production to be achieved if given the same space, as required by 12,650 solar panels. With the results obtained, the share of energy demand covered by the WECs is estimated comparing it to that generated by other renewable energy sources.

Section 2 describes the study area. Section 3 shows the available data and the methodology used to quantify the wave resource. Section 4 estimates the average energy power (AEP) for two different WEC at the chosen locations nearby the Port of Valencia and Section 5 discusses the results obtained in Section 4. Finally, Section 6 presents the conclusions of the paper.

## 2. Data and Methodology

### 2.1. Study Area

**Figure 1:**
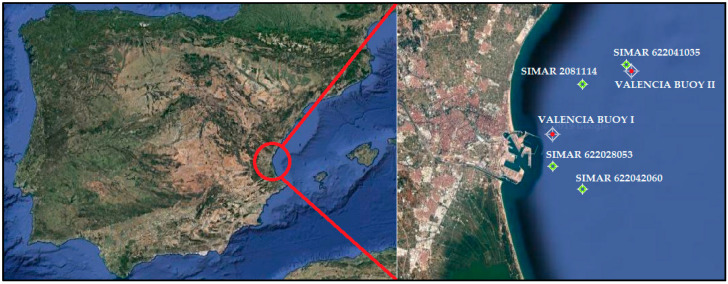
Location of the Port of Valencia in the western Mediterranean Sea (**left**). Location of the analysed buoys and points (**right**).

The study area is located in the western Mediterranean Sea (39.45° N—39.40° N, 0.31° W—0.20° W) (Figure 1).

The Port of Valencia has a total surface of 5,626,534 m^2^ [14]. The main cargo handled within the Port of Valencia is containerized, with over 5 million (Mio) TEU (Twenty feet Equivalent Units) handled in 2018. This means that the majority of the port’s surface is dedicated to container handling. The Port of Valencia has three container terminals covering a total surface of over 2 million m^2^. These three terminals together with a terminal for refrigerated cargo are the main electric energy consumers of the port, with over 80% of the consumption overall [15].

The climate in the Bay of Valencia is influenced by the semi-arid climate of mid-latitudes. This means that it is dominated by extra-tropical cyclones formed through baroclinic instability, which is at its highest during the winter season. In addition, it is affected by depressions in movement generated in the Atlantic Ocean or in north-western Europe [16]. According to [17], Mediterranean storms are usually shorter and less intense than those in northern Europe, with many sub-regional and mesoscale effects that produce great spatial and seasonal variability [18]. The reduced scale, together with the peculiar characteristics of the basin (the complex orography and the humidity of a relatively large body of water) makes the Mediterranean climate more difficult to predict than climates elsewhere [19]. During the summer season, thermal and orographic effects play a more important role in the genesis and maintenance of cyclones. In the north-western Mediterranean, the main cyclone centres are located again in the Gulf of Genoa and in the Iberian Peninsula, the latter caused by the contrasts of temperature between land and sea [18]. During the warm periods, the Mediterranean is also exposed to tropical systems [20] as a result of its location in a transition zone between humid mountains in the north and arid regions in the south. Finally, spring and autumn can be considered periods of transition between the contrasting patterns of winter and summer [20].

The Mediterranean Sea represents, in terms of wave energy power availability, an intermediate level between the open ocean and the enclosed small-fetch basins such as the Black Sea or the Baltic Sea. Hence, wave energy exploitation seems to be promising even if the net quantities are not as significant as in the open ocean [21].

### 2.2. Available Wave Data

Wave Modelling (WAM) is a third generation model that integrates the basic transport equation that describes the evolution of a two-dimensional ocean wave spectrum without additional unplanned assumptions regarding spectral shape. There are three explicit source functions that describe wind input, non-linear transfer and white-capping dissipation. There is an additional source function for background dissipation and the refraction terms are included in the finite depth version of the model. The model runs in a spherical grid of latitude and longitude and can be used in any ocean region.

WAM predicts the directional spectra along with wave properties such as significant wave height, wave direction and mean wave frequency, wave height and mean wave direction, and corrected wind stress fields including wave-induced stress and drag coefficient at each point in the grid at the chosen output times.

WAM can be coupled to a range of other models. Examples include the Southeast Asian Ocean Model (SEAOM), the Coastal Ocean Modelling System of the Proudman Oceanographic Laboratory (POLCOMS), the Core Model for European Ocean Modelling (NEMO), the High-Resolution Limited Area Model (HIRLAM), and the Regional Climate Model (RegCM) of the National Centre for Atmospheric Research (NCAR) [22].

The Spanish Ports Authority (Puertos del Estado) has developed and implemented a two-way nesting procedure in the model for the Spanish coast. Using this system, the equation is integrated at the same time for all points. Since it is possible to define the spacing depending on the grid point location, it works as a variable spacing schema. The resolution is enhanced using intermediate grids, which are placed between the coarse and the fine grids.

In the particular case of the Mediterranean domain, the shallow water version of the WAM model is used, therefore, refraction and attenuation effects are considered for those (few) grid points located in shallow waters [23].

This model produces the wave directional spectra for each grid point. Then, it is used to obtain further information, i.e., Hs (significant wave height), Tp (peak wave period), Tm (mean wave period), mean direction, wind sea and swell components, etc.

The WAM model obtained information that is made available for the Bay of Valencia from the wave climate database stored by Puertos del Estado. This database contains data of 7-year hindcast wave climate using the WAM model and forced by the wind output of the HIRLAM regional atmospheric model. Data are taken from three points within the Bay of Valencia. However, a comparison between the data given by these points, obtained from a numerical simulation, with the real data obtained by the Buoy of Valencia II (0.20° W—39.51° N) from the same time period must be made in order to validate the data given by the WAM model.

### 2.3. Methodology

**Figure 2:**
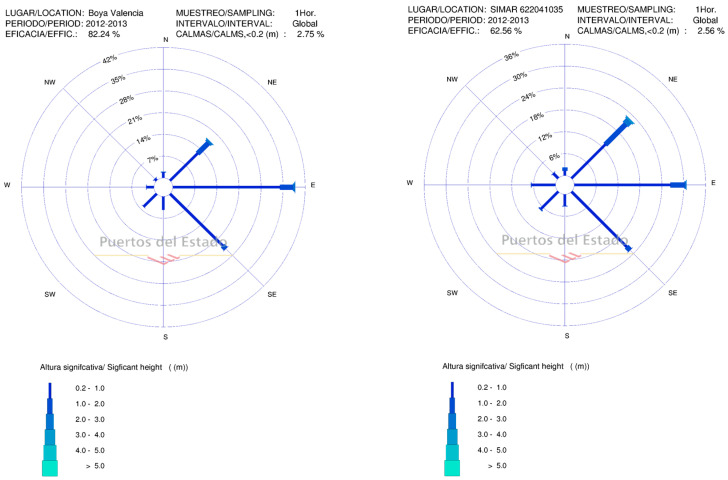
Wave rose: Valencia Buoy II (**left**). SIMAR 622041035 (**right**). Period 2012–2013.

First of all, the validation of the SIMAR data must be granted. This validation lies in the work of Puertos del Estado [24,25]. In addition, the similarity of the data can be shown by looking at the Figure 2 below that is from a period of time in which both systems were working (2012–2013). This Figure 2 shows the wave rose according to both the Valencia buoy II and the SIMAR 622041035.

The period of 2012–2013 has been selected because it is the only period in which data from both the Valencia Buoy II and the SIMAR 622041035 are recorded in the Puertos del Estado database. After 2013 the Valencia Buoy II was dismantled.

As a preliminary conclusion, the data from both the buoy and the SIMAR 622041035 are alike and not much difference is noted between them. From now onwards, the available wave resource will be estimated by using the SIMAR points when we refer to the area around the Port of Valencia, as there are no available data from any buoys.

Both the Valencia Buoy and the SIMAR 6220241035 show a predominant wave direction from the E, together with other contributions from the NE and SE.

Considering Table 1 and Table 2, it is deduced that for the Valencia Buoy II, 86% of the waves have a Hs of 1 m or less while 85% of them have a Tp of 7 s or less. In the case of the SIMAR, there is the same range of Hs and Tp, 86% and 76% of frequency are obtained, respectively. These results represent no deviation for the Hs and a 9% for the Tp, which could be acceptable according to [25,26,27,28].

Once the origin of the data is defined, using the following equations, the power resource can be estimated.

All points considered are located in deep water (50 m), therefore the propagation processes such as refraction and diffraction can be neglected. Wave power estimation can be calculated with the following deep-water equation:

(1) $$P=\;\frac{\rho g^{2}}{64\pi }Hs^{2}Te\;≅0.491Hs^{2}Te$$

where *P* is the wave power per unit of crest length (kW/m), *Hs* is the significant wave height, *Te* is the energy period, *ρ* is the density of seawater (assumed to be 1025 kg/m^3^) and *g* is the gravitational acceleration.

As pointed out in [29], one approach when *Tp* is known is to assume the following:

*Te = αTp* (2)

A value of *Te* = 0.9 *Tp* [30] was used to assess the wave energy resource, estimating *P* using Equation (2).

(3) $$P≅0.442Hs^{2}Tp$$

## 3. Analysis of Wave Energy Resource

The aim of this research is, apart from estimating the available wave resource in the study area, to propose different alternatives of marine energy generators that can meet the infrastructural requirements of a port such as the Port of Valencia. Special focus is made on those able to produce energy from waves. In this case, it is considered that the data showing the state of the sea of the SIMAR 622028053 provides more information about the actual sea state at the Port of Valencia than the data given by the other possible SIMAR points, as its location is a better match with the potential location of the WECs. Therefore, from now on the data obtained from this SIMAR 622028053 will be considered for the purpose of this study.

Using the previous Equation (2), and the data given by the Puertos del Estado database [24], for the SIMAR 622042060, SIMAR 2081113 and SIMAR 622028053 during the period of 2012–2019, the results shown in Table 3 can be obtained.

Where Tpav, Hsav and Pw are the average values for Tp, Hs and Pw for the 2012–2019 period at the selected SIMAR points and buoys and Pw the wave power.

The seasonal wave power fluctuation at the Valencian Bay will now be analysed. With the purpose of having more detailed information about the variability of the resource, the seasonal variability (SV) index is introduced and defined as the difference of the most energetic season minus the lesser one divided by the yearly average value evaluated throughout the whole data set [31]:

(4) $$SV=\;\frac{P_{Smax}-P_{Smin}\;}{P_{Year}}$$

where *P_Smax_* is the mean wave power for the highest-energy season and *P_Smin_* is the mean wave power for the lowest energy season, and *P_year_* is the annual mean wave power. The greater the value of *SV* the larger the seasonal variability, with values lower than 1 indicating moderate seasonal variability.

We will now analyse the SV for the two boys close to the study area as there are no data available for the SIMAR 622028053.

In our case, considering the information from Puertos del Estado for the Valencia Buoy II, available at [24], *P_Smax_* (winter) = 6.62 kW/m and *P_Smin_* (autumn) = 5.79 kW/m, we obtain, thus, a result of *SV* = 0.17. However, if we take the same data from the Valencia Buoy I, *P_Smax_* (winter) = 7.04 kW/m and *P_Smin_* (spring) = 4.79 kW/m, we obtain, thus, a result of *SV* = 0.46. As we have considered for the purpose of this paper the data obtained from SIMAR 622028053 whose location is closer to the Valencia Buoy I, we should consider the seasonal variability of the latter instead.

**Figure 3:**
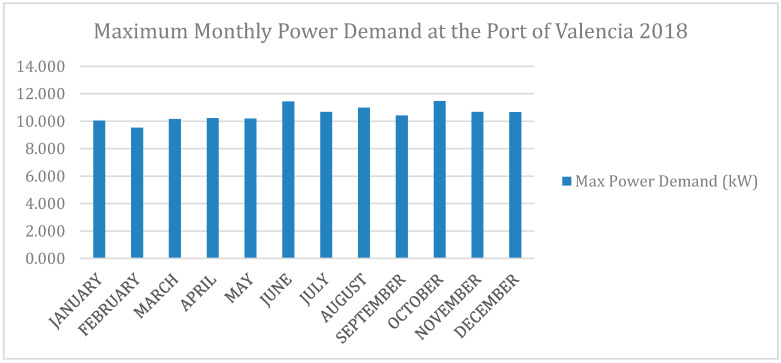
Maximum monthly power demand at the Port of Valencia in 2018 (Source: Port Authority of Valencia).

The above results mean that there is very low seasonal variation for the wave resource in the study area, which would be a good starting point if the monthly variation of energy demand is considered at the Port of Valencia (Figure 3).

**Figure 4:**
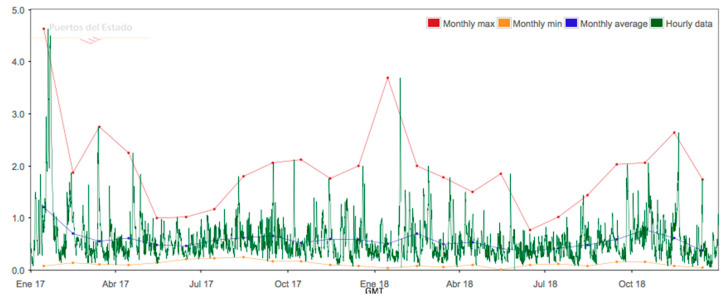
SIMAR 622028053 wave significant height 2017–2019.

**Figure 5:**
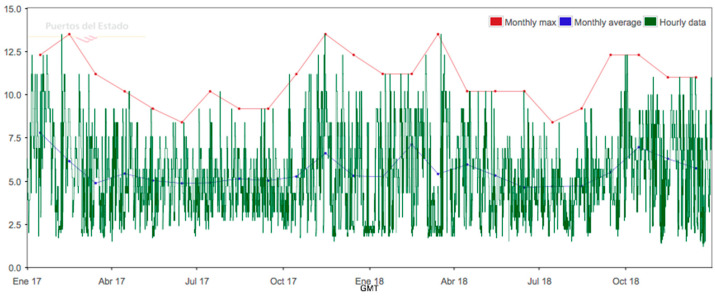
SIMAR 622028053 peak period 2017–2019.

**Figure 6:**
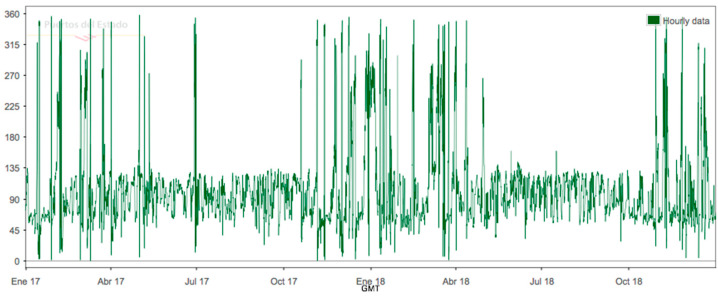
SIMAR 622028053 predominant wave direction 2017–2019.

Only data from a two-year interval have been provided by the Puertos del Estado database. There are no data for longer than two years. The variation of Hs, Tp and direction for 2017–2019 period is shown in Figure 4, Figure 5, and Figure 6.

Several studies have provided analysis of potential production and estimated performance of different WEC technologies [32,33,34,35,36,37,38].

For a sea state like that described in Table 4 the average energy production (AEP) could be estimated for two types of WEC suitable to be integrated into the port’s breakwater [39,40]. The first system is the Eco Wave Power and the second is the Overtopping Breakwater (OTD) for Wave Energy Conversion (OBREC). This election is based on the discussion shown in [7], as these two systems are easily embedded in the port’s breakwaters.

The choice of these systems is based on: (a) the development stage of both technologies, running real tests, one of them being grid connected, (b) the manufacturers of both technologies have made their forecasted power matrices available for the purpose of this study, (c) the WECs have different operating principles and (d) both technologies are suitable for the integration of the devices into a port’s infrastructure.

The characteristics of these two systems are explained below.

**Figure 7:**
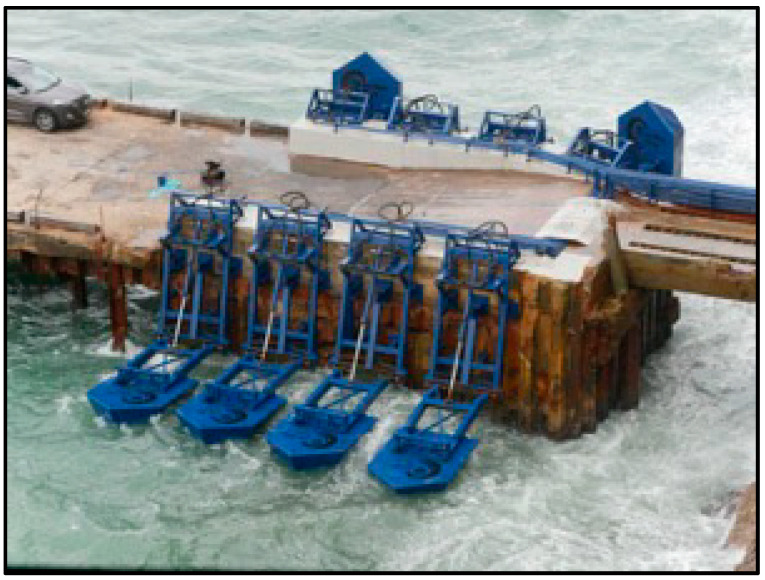
Eco Wave Power system in Gibraltar. Source: www.ecowavepower.com.

The Eco Wave Power located in Gibraltar (Figure 7) comprises 8 floaters that draw energy from incoming waves by converting the rising and falling motion of the waves into a clean energy generation process. Each floater is linked by a robust arm to a jetty and has the shape of a rectangular box and a surface area of about 4.68 m^2^.

**Figure 8:**
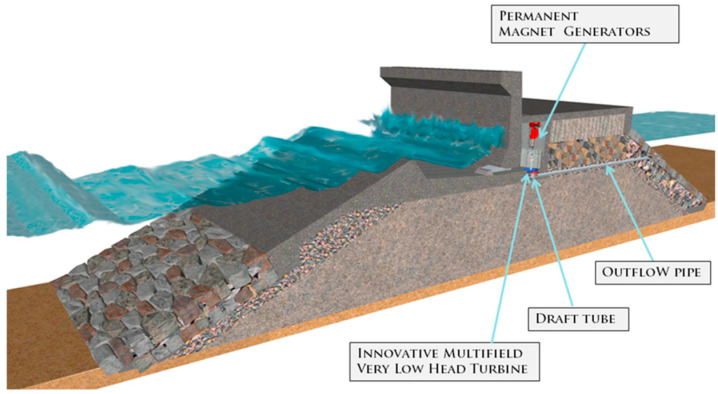
Lateral view of the OBREC device in Italy (Reprinted from Sustainability, Contestabile et al., 2016).

The second device is OBREC. This system is integrated in a traditional breakwater and can be considered an innovative non-conventional breakwater (Figure 8). It fulfils the same functions as traditional breakwaters with the added value of power generation. This technology captures part of the wave run-up on the slope of the device. The potential energy of the stored water in the reservoir is converted into kinetic energy, flowing through low head turbines located in an engine room behind the reservoir. The energy is, thus, converted into electrical energy by means of generators coupled to turbines. It fulfils the same functions as traditional breakwaters in terms of coastal protection with the added value of power generation. It is the first overtopping WEC totally integrated into an existing breakwater.

## 4. Results

As mentioned in the previous section, the AEP of the WEC devices can be calculated by combining the wave energy resource characterization matrix in the study area with the power matrices of the devices.

According to the Eco Wave Power calculation method, wave heights and period on the site, and considering a potential location on the Valencia breakwaters, Eco Wave Power has forecasted the possibility of installation of up to 70 floaters on the external side of the breakwater, with 0.5 spacing, and with a surface area of 20 m^2^ for each floater. This kind of arrangement will enable an installed capacity of 300 kW, with an AEP of 0.8 GWh/year and with a capacity factor of 28.8 %, which is higher than the capacity factor available by solar energy and equal to the capacity factor to be achieved by installing wind turbines (see Table 5, showing the capacity factor for solar is 18%). However, considering the scaling potential of the Eco Wave Power system and an available breakwater length of 1000 m the AEP would be in the region of 2.6 GWh/year. In comparison with that, the photovoltaic plant would need 26,565 square meters for an installed power of 5.5 MW with an estimate of production of 10.1 GWh/year considering a capacity factor of 21%, which is 18.7% of the energy consumption by the Port of Valencia.

**Figure 9:**
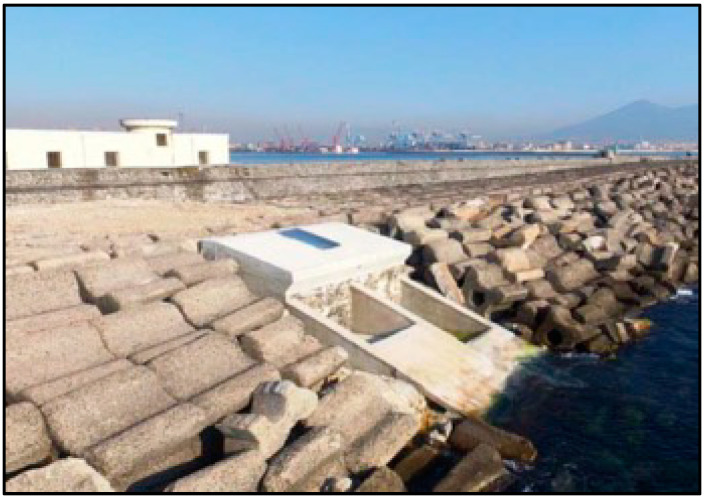
Lateral view of the OBREC device in Italy (Reprinted from Sustainability, Contestabile et al., 2016).

On the other hand, a definite power matrix of the OBREC is not available yet, since the first full-scale prototype installed at Naples Harbour (Italy) is currently under monitoring (Figure 9); therefore, for the purpose of this paper and based on previous research [40], considering that the device could be integrated along the designed length of the new breakwater (500 m), and based on the aforementioned research, the AEP that could be provided by the OBREC would be in the region of 2.5 GWh [40,41,42,43]. However, this type of technology cannot be installed on each meter as some spacing and energy lost in conversion should be considered. In that case the capacity factor of the OBREC would be estimated at around 15% [44], much less compared to that from Eco Wave Power.

In the case of Valencia, however, with a breakwater length of 500 m, calculations for the OBREC gave an estimation of around 1.60 GWh/year of energy production (EnerOcean, personal communication, 26 July 2019), a little lower than the estimates given by previous research that reached AEPs of 3.5 GWh for Imbituba, Brazil and 2.5 GWh for San Antonio, Chile [45] but in line with the expected production considering the poorer wave resource of Valencia compared to both aforementioned locations. 

As some researchers have already stated [46], OBREC devices give competitive energy outputs when 0.5 < Hs < 1 and 7 < Tp < 8 [47] which, apparently, is a better match with the wave resource available in the study area. 

This condition occurs only 20% of the year in the case of the Bay of Valencia. This fact would be an advantage for the Eco Wave Power System as it could work at any wave height and period.

For the Valencia sea conditions previously set and the simulated performance of the two selected WECs, 0.8 GWh/year and 1.6 GWh/year could be expected, which means, according to the electricity consumed by the Port of Valencia in 2016 given by [48], around 3% of the electricity needs overall. However, and due to the scaling potential of the Eco Wave Power technology, if an array of 1000 on the Port of Valencia’s breakwater is installed, an energy production of 2.6 GWh/year is estimated.

Considering that the CO_2_ equivalent to the electricity used by the Port of Valencia in 2016 was 18,392 tCO_2_eq, using the WEC technologies, around 500 tCO_2_eq could be avoided per year in the worst scenario.

## 5. Discussion

Extracting energy from waves is a promising solution to cover part of a port’s energy demand. In addition, this solution is carbon-free and can be combined with other carbon-free energy sources such as wind or solar.

Port infrastructure could be designed to accommodate new facilities able to generate energy from renewable sources such as the ones mentioned above. Considering the wave energy converters, we should choose those capable of performing two tasks at the same time: producing energy and protecting the coastline from the effects of climate change.

When assessing the possibility to use a port’s infrastructure for the installation of wave energy converters, some of the topics related to the climate conditions at the test site should be considered: the orientation of the breakwater with respect to the incident waves, geographical location and wave stability. Other issues to be considered are technical issues: PTO efficiency, cost effectiveness, scalability, grid connectivity, environmental impact and logistics. In addition, when considering a project as a whole, all costs and risks involved should be taken into account and the efficiency is only part of the equation while other factors, such as maintenance and operating costs or environmental issues, among others, could make one project a better choice than another.

Some of these technologies are not at an optimal technology readiness level (TRL) yet, however, we have experiences of facilities presently working close to the study area giving promising results. For example, Eco Wave Power has had an installed and grid connected power station in Gibraltar since 2016 that is not far from feasible TRL, and the OBREC system is installed in a breakwater at the Port of Naples. The architecture of some of these WECs make them capable of giving reasonable protection to coastal environments, as these devices could provide both power generation and coastal protection, one could be compensated by the other [4].

As for coastal protection, a terminator WEC, such as the OBREC, would be the best option, as it can better dissipate the energy of the incoming waves [40], although for the purpose of this paper a point absorber near-shore system is also considered, especially given the pointed design of the Eco Wave Power floaters, which cut through the waves, thus decreasing some of the erosive effects of the waves.

By deploying WECs the significant wave heights breaking at a certain location can be modified [40], and this is a positive factor that could support the success of a project. Therefore, when assessing the commissioning project of a WEC array, it is important to assign a monetary value to the ecological effects, the coastal protection action and the emissions of greenhouse gases and other air pollutants that have been avoided.

Two WEC systems particularly capable of being embedded in a port’s breakwater have been chosen to evaluate the AEP from the available wave resource at the Valencia Bay.

Both systems are scalable but different in their architecture, the OBREC being part of the breakwater infrastructure while the Eco Wave Power system must be linked to it. The results obtained for these two systems for the sea condition of the Valencia Bay are that far in preliminary assumptions. In terms of efficiency, the Eco Wave Power’s efficiency is close to 50%, and the capacity factor is almost 29% in the aforementioned configuration, while the efficiency of the OBREC is around 15% according to the estimates in the case study.

The results previously obtained for the WECs can be compared to the results of the evaluation studies of energy production of wind and solar origin, carried out by the Valencia Port Authority.

In the case of the wind energy assessment, and according to the previous studies, the estimated installed power in the port of Valencia would be around 15 MW. To meet this requirement, various wind turbines between 3 MW and 4.8 MW nominal power could be placed. According to the data obtained from the meteorological network located in the Port of Valencia, the average wind resource available would be about 5.2 m/s. By introducing this data in the different performance curves of the suitable wind turbines together with the aforementioned limitations, a capacity factor between 26% and 30% would be obtained. Considering the most pessimistic estimates, the operating hours of the wind turbine would be around 2300 h, obtaining an AEP of approximately 27 GWh/year.

On the other hand, when considering the installation of solar panels, the port of Valencia has free surfaces on the roof of a new vehicles storage silo capable of holding approximately 12,650 panels. Considering a panel type of 435 W/unit, its capacity factor in the latitude of the Port of Valencia is about 18%, and therefore the estimate AEP would be in the order of 8 GWh/year.

There are references to the capacity factor of different technologies, including those that produce energy from renewable sources in Table 5 [49].

Most of the estimated CF for the three evaluated technologies in the study area are in line with those shown in Table 5 for the UK, except for the solar technology which is a bit higher for the location in Valencia, and wave energy, which is at 29% in the case of the Eco Wave Power technology.

Consequently, the installation of renewable energy sources (including the ocean) is feasible if the amount of energy production is not the key factor for the decision-making process. All the three potential energy sources considered above supply an amount of energy capable of covering more than 50% of the current energy needs of the Port of Valencia [7].

The great advantage of the Eco Wave Power system in contrast to the OBREC one is that the former is highly scalable, the cost is much lower, and the maintenance operations are quite simple while the latter is fully integrated into the breakwater and has less margin in case of failure or scaling needs.

Further research has to be done to improve the performance of the WEC devices. For example, other authors have designed a hybrid system that combines the OTD and OWC technologies, that could eventually improve the system performance in terms of energy production, however there are no available data in this respect [50].

## 6. Conclusions

It is a fact that for some years now ports have been adapting their infrastructures to face the challenges posed by the energy transition and climate change. There is an increasing number of examples of ports that are progressively integrating renewable energy (and marine energies) into their facilities with the double objective of producing clean energy to meet their needs and protecting their infrastructure from the effects of climate change.

This paper evaluates the potential integration of renewable marine energies in port infrastructure, with special focus on the estimation of the wave energy resource at the Bay of Valencia, near the Port of Valencia. The purpose of this paper is to estimate the wave energy resource by simulation tools using the WAM model forced by the wind output of the HIRLAM regional atmospheric model, and to study of the performance of two WECs capable of being integrated into the port’s shelter infrastructure.

Among the different technologies of WEC, those categorized as near-shore would be the best option for the purpose of this paper. This assumption is reached considering as primary selection criteria the existence of a relatively exposed deep-water breakwater, such as those existing in ports. Due to the early stage of development, a reliable system should be implemented, that can be granted in ports, where a direct-current transmission can be obtained and low maintenance costs are involved, compared to those for offshore WECs.

Research has been undertaken among the different technological developers and two potential WECs have been identified: the OBREC and the Eco Wave Power systems, respectively.

The OBREC estimated AEP is relatively low, and the Eco Wave Power’s AEP is comparable to solar, due to the fact that the available wave resource in the study area is poor, but this could be compensated by the fact that logistics, operation and maintenance costs are much lower, and coastal protection performance is much higher than those of the offshore WECs and other power generators based on different energy sources such as wind or solar.

At this point, a decision must be made considering the installation of renewable energy generators in ports. Although ports have large surfaces, these surfaces are not really made to accommodate energy generators as they do not produce large profits. On the other hand, a price should be set on the new climate conditions created by climate change. We put in the same equation all these variables; cost of energy, cost of missing biodiversity, higher flood risks and consequently economic loss in port terminals, revenues by exploiting the port infrastructure, etc.

In addition, although there is no ideal technology, we should opt for those that allow a greater use of the resource, adjusting more to the wave regime that is most often present in the study area. More pilot tests would be needed with the acquired knowledge from previous locations and the technological development from these experiences.

It is a fact that wind and solar energy technologies are at a very high level of maturity although there is still room for further improvement. By contrast, further research should be conducted in the case of wave power generators capable of being integrated into ports’ infrastructure. This research should focus on hybrid solutions that combine several renewable sources, such as wind and waves or tides and waves or wave and solar, such as the new patent submitted by Eco Wave Power, which incorporates solar panels on top of the floaters, thus saving space, while providing an additional energy source. In this case, the design of these energy devices should be considered when designing ports’ shelter structures.

## Figures and Tables

**Table 1:** Hs (significant wave height) vs. Tp (peak wave period) table Valencia Buoy II.

Year 2005–2013	Tp (s)												
		≤1.0	2.0	3.0	4.0	5.0	6.0	7.0	8.0	9.0	10.0	>10.0	TOTAL
	≤0.5	--	---	3.870	8.883	9.467	8.688	6.514	2.029	0.267	0.160	0.066	39.946
	1.0	---	---	3.747	8.944	12.058	7.647	6.834	5.199	1.171	0.237	0.015	45.854
	1.5	---	---	---	0.360	1.940	2.158	2.040	1.880	0.911	0.533	0.047	9.869
	2.0	--	---	---	0.002	0.103	0.481	0.697	0.694	0.360	0.405	0.059	2.798
	2.5	---	---	---	---	0.003	0.056	0.246	0.302	0.160	0.142	0.042	0.952
Hs (m)	3.0	---	---	---	---	---	0.002	0.033	0.145	0.053	0.070	0.035	0.337
	3.5	---	---	---	---	---	---	0.012	0.098	0.035	0.023	0.006	0.174
	4.0	---	---	---	---	---	---	---	0.029	0.017	0.011	0.003	0.059
	4.5	---	---	---	---	---	---	---	---	0.003	0.005	0.005	0.012
	5.0	---	---	---	---	---	---	---	---	---	---	---	0.000
	>5.0	---	---	---	---	---	---	---	---	---	---	---	0.000
	TOTAL	---	---	7.617	18.188	23.571	19.031	16.377	10.376	2.977	1.585	0.278	100%

**Table 2:** Hs vs. Tp Table SIMAR 622041035.

Year 2005–2013	Tp (s)												
		≤1.0	2.0	3.0	4.0	5.0	6.0	7.0	8.0	9.0	10.0	>10.0	TOTAL
	≤0.5	---	4.931	9.735	10.245	6.878	7.461	6.405	1.710	1.137	0.801	0.391	49.695
	1.0	---	---	1.456	5.350	6.296	5.714	7.679	4.176	2.184	1.410	2.156	36.421
	1.5	---	---	---	0.127	1.065	1.092	1.219	1.337	1.638	0.901	1.747	9.126
	2.0	---	---	---	---	0.064	0.209	0.191	0.209	0.582	0.464	1.028	2.748
	2.5	---	---	---	---	---	0.009	0.146	0.064	0.155	0.246	0.355	0.974
Hs (m)	3.0	---	---	---	---	---	---	0.082	0.027	0.191	0.200	0.045	0.546
	3.5	---	---	---	---	---	---	0.018	0.073	0.082	0.100	0.100	0.373
	4.0	---	---	---	---	---	---	---	---	0.009	---	0.073	0.082
	4.5	---	---	---	---	---	---	---	---	---	---	0.036	0.036
	5.0	---	---	---	---	---	---	---	---	---	---	---	0.000
	>5.0	---	---	---	---	---	---	---	---	---	---	---	0.000
	TOTAL	---	4.931	11.191	15.722	14.303	14.485	15.740	7.597	5.978	4.122	5.932	100%

**Table 3:** Average values of Tp and Hs and estimation of the Pw for the SIMAR points around the Port of Valencia.

Data Source	Tpav (s)	Hsav (m)	Pw (kW/m)
SIMAR 622042060	5.94	0.93	5.67
SIMAR 2081113	6.85	0.85	5.12
SIMAR 622028053 Valencia buoy I (*) Valencia buoy II (**)	5.96 5.98 5.58	0.84 0.82 1.10	4.99 4.85 5.51

(*) Valencia buoy I was working during the 1985–2005 period. (**) Valencia buoy II was working during the 2005–2013 period.

**Table 4:** Hs vs. Tp Table SIMAR 622028053.

Year 2012–2019	Tp (s)												
		≤1.0	2.0	3.0	4.0	5.0	6.0	7.0	8.0	9.0	10.0	>10.0	TOTAL
	≤0.5	---	3.170	8.712	10.982	8.249	8.606	6.023	1.794	1.226	0.857	0.432	50.051
	1.0	---	---	1.397	5.184	6.962	6.641	8.340	3.504	2.420	2.159	1.441	38.048
	1.5	---	---	---	0.107	0.897	1.271	1.199	1.009	1.075	1.110	1.497	8.165
	2.0	---	---	---	---	0.030	0.243	0.320	0.184	0.316	0.434	0.740	2.267
	2.5	---	---	---	---	---	0.012	0.149	0.068	0.093	0.173	0.255	0.750
Hs (m)	3.0	---	---	---	---	---	---	0.024	0.072	0.094	0.084	0.061	0.335
	3.5	---	---	---	---	---	---	0.003	0.049	0.072	0.070	0.040	0.234
	4.0	---	---	---	---	---	---	---	0.002	0.014	0.042	0.028	0.086
	4.5	---	---	---	---	---	---	---	---	0.007	0.005	0.044	0.056
	5.0	---	---	---	---	---	---	---	---	---	0.005	0.003	0.008
	>5.0	---	---	---	---	---	---	---	---	---	---	---	0.000
	TOTAL	---	3.170	10.109	16.273	16.138	16.773	16.058	6.682	5.317	4.939	4.541	100%

**Table 5:** Capacity factors in 2013 for various UK power plants. Data from the Department of Energy and Climate Change (DECC).

Plant Type	Capacity Factor (%)
Nuclear power plants	73.8
Combined cycle gas turbine stations	27.9
Coal-fired power plants	58.4
Hydroelectric power stations	31.7
Wind power plants	32.3
Photovoltaic power stations	10.2
Marine (wave and tidal power stations)	9.7
Bioenergy power stations	58.0

## Abbreviations

AEP	Annual Energy Production
AWP	Autonomous Wave Prediction System
CF	Capacity Factor
HIRLAM	High Resolution Limited Area Model
OBREC	Overtopping Breakwater for wave Energy Conversion
OTD	Overtopping Devices
OWC	Oscillating Water Column
PAV	Port Authority of Valencia
PTO	Power Take Off
SV	Seasonal variability
TEU	Twenty Feet Equivalent Unit
TRL	Technology Readiness Level
WAM	Wave prediction Model
WEC	Wave Energy Converters
